# Terahertz Photons Improve Cognitive Functions in Posttraumatic Stress Disorder

**DOI:** 10.34133/research.0278

**Published:** 2023-12-15

**Authors:** Yun Yu, Kaijie Wu, Xiao Yang, Jiangang Long, Chao Chang

**Affiliations:** ^1^ School of Life Science and Technology, Xi’an Jiaotong University, Xi’an 710049, China.; ^2^ Innovation Laboratory of Terahertz Biophysics, National Innovation Institute of Defense Technology, Beijing 100071, China.; ^3^ School of Physics, Peking University, Beijing 100871, China.

## Abstract

Posttraumatic stress disorder (PTSD) is a serious psychosis leading to cognitive impairment. To restore cognitive functions for patients, the main treatments are based on medication or rehabilitation training but with limited effectiveness and strong side effects. Here, we demonstrate a new treatment approach for PTSD by using terahertz (THz) photons stimulating the hippocampal CA3 subregion. We verified that this method can nonthermally restore cognitive function in PTSD rats in vivo. After THz photon irradiation, the PTSD rats’ recognitive index improved by about 10% in a novel object recognition test, the PTSD rats’ accuracy improved by about 100% in a shuttler box test, the PTSD rats’ numbers to identify target box was about 5 times lower in a Barnes maze test, and the rate of staying in new arm increased by approximately 40% in a Y-maze test. Further experimental studies found that THz photon (34.5 THz) irradiation could improve the expression of NR2B (increased by nearly 40%) and phosphorylated NR2B (increased by about 50%). In addition, molecular dynamics simulations showed that THz photons at a frequency of 34.5 THz are mainly absorbed by the pocket of glutamate receptors rather than by glutamate molecules. Moreover, the binding between glutamate receptors and glutamate molecules was increased by THz photons. This study offers a nondrug, nonthermal approach to regulate the binding between the excitatory neurotransmitter (glutamate) and NR2B. By increasing synaptic plasticity, it effectively improves the cognitive function of animals with PTSD, providing a promising treatment strategy for NR2B-related cognitive disorders.

## Introduction

Posttraumatic stress disorder (PTSD) is the most common psychopathological consequence of exposure to traumatic events, whose prevalence can be as high as 50% in mental health facilities [1]. One important symptom of PTSD is the cognitive dysfunction [2–4], and the primary clinical treatment for PTSD involves the restoration of patients' cognitive function. Therapies for PTSD include psychological, pharmacological, and innovative interventions. Trauma-focused cognitive behavioral therapy is the most effective psychological intervention for PTSD. However, it is worth noting that this treatment may not yield positive outcomes for approximately 50% of patients [3,5]. Currently, the only Food and Drug Administration-approved medications for PTSD are sertraline and paroxetine, which are serotonin reuptake inhibitors. However, these medications often target only specific aspects of the disorder or may be effective for only a subset of patients, failing to sufficiently improve cognitive function [2,6,7]. Therefore, there is a pressing need to explore novel and effective therapeutic modalities that can restore cognitive function in individuals suffering from PTSD.

The hippocampus is a crucial component of the limbic system and plays an important role in the formation of spatial memory, the consolidation of short-term memory, and the formation of long-term memory. The initial encoding of hippocampal memories is thought to be implemented via plasticity of feedforward mossy fiber synapses onto CA3 pyramidal cells and at recurrent pyramidal cell synapses [8,9]. Thus, enhancing the neuronal function within the CA3 region can lead to improvements in learning and memory abilities.

N-methyl-D-aspartate receptors (NMDARs) are expressed throughout the central nervous system (CNS) and play key physiological roles in synaptic function, such as synaptic plasticity, learning, and memory. NMDARs are heterothermies composed of NR1, NR2A, and NR2B subunits, which bind glycine and glutamate, respectively [10,11]. GluN2 could be activated by binding L-glutamate and relieving a magnesium block of the ion channel pore by membrane depolarization [12]. NMDARs are also implicated in the pathophysiology of several CNS disorders and more recently have been identified as a locus for disease-associated genomic variation [11,13]. Therefore, using agonists or even improving the expression of NMDARs to enhance their function is expected to be helpful for learning and memory in the CNS.

The frequency domain for the response of living organisms consistently lies in the spectrum ranging from 0.1 to 100 terahertz (THz) [14]. Within this frequency range, THz photons exhibit multitude biological applications [14]. For cancer treatment, 83-THz photon irradiation can inhibit the migration of tumors both in vitro and in vivo [15]. In the nervous system, the 53.6-THz photons can also regulate the activity of brain nerves and increase the learning speed of mice by 50% [16]. It has also been shown that THz photons can affect ion channels and enhance voltage-gated currents to accelerate the repolarization of action potentials, thereby shortening the action potential photons form and regulating the startle response of zebrafish [17,18]. The evidence further demonstrates that specific-frequency THz photons possess the ability to modulate chemical synaptic transmission and alter neuronal signal emission in vitro [19]. However, the THz photons could be intensely absorbed by tissue water, preventing THz arriving at the target inside the body.

**Fig. 1.**
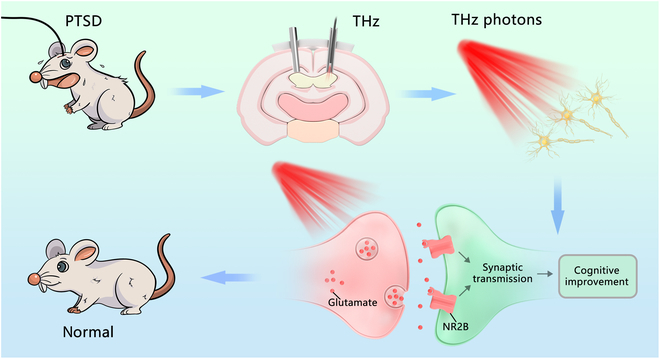
Schematic illustration of in vivo effect of THz irradiation on cognitive function in PTSD rats. THz photon irradiation of hippocampal CA3 subregion (the irradiated coordinates were [±1.5 mm, −2.16 mm, −3.5 mm]) could improve the cognitive function of animals with PTSD. By enhancing glutamate binding to NR2B in the CA3 subregion, synaptic plasticity was enhanced, ultimately restoring the cognitive function in PTSD rats.

In this paper, we presented an innovative methodology, named as deep brain terahertz stimulation (DBTS), aimed at enhancing cognitive function in individuals with PTSD. The advantages of DBTS include: the essential characteristic THz interaction and response of biomolecules compared to the optogenetics technique requiring exogenous genes restricted from applications in healthy humans, the precise stimulation in millimeter level compared with the dispersion of electric and magnetic technique, and achieving the optimized THz frequencies for different bioeffects by scanning various frequencies. The DBTS methodology involved the precise implantation of a metal tube at the specific target site requiring irradiation. By precisely coupling the THz photons emitted from quantum cascade laser (QCL) into a low-loss THz fiber, we employed a stereotaxic apparatus to insert the fiber directly through the metal tube, reaching the designated deep brain region (hippocampus CA3 subregion) with utmost precision (Fig. 1). In contrast to previous techniques that involved exposing the entire animal to THz photons or irradiating the animals externally through the skin [20,21], our approach allowed for much more accurate and targeted irradiation of the desired deep brain region.

## Results

### In vivo experiments showed that THz photons (34.5 THz) irradiation of the CA3 subregion reversed the learning and memory dysfunction of PTSD rats

**Fig. 2.**
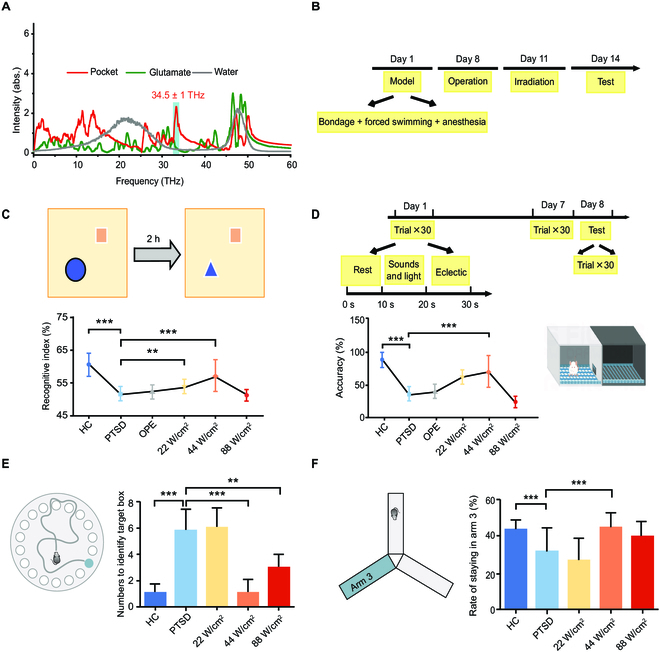
In vivo effect of THz photons (34.5 THz) irradiation on cognitive function in PTSD rats. (A) The absorption spectra of the glutamate-binding pocket of the NR2B receptor (red curve), glutamate molecule (green curve), and water molecule (gray curve) were calculated using molecular dynamics simulations. (B) Schematic diagram of experimental procedure and establishment of PTSD rats. (C) Results of novel object recognition test. Recognitive index was calculated as time investigating novel object / (total time investigating novel and old objects). (D) Results of shuttle box test. Accuracy means number of positive avoidance / total number of exercises. (E) Results of Barnes maze test. (F) Results of Y-maze test. HC, health control group; PTSD, PTSD model group; OPE, sham-operated group. (All data shown as means ± SD, **P < 0.01; ***P < 0.001.)

NR2B is an essential subunit of NMDARs, plays a crucial role in neuronal excitability, and is associated with the learning and memory function of the nervous system. Increased expression or activation of NR2B could revert the learning and memory abilities of mice with impaired cognitive function [22,23]. To confirm the details of THz photon absorption, we calculated from molecular dynamics simulations that the absorption spectrum of the glutamate-binding pocket of NR2B shows a clear fingerprint peak near 34.5 ± 1 THz, while this main absorption peak was located for glutamate molecules (Fig. 2A). Notably, the THz photons corresponding to frequencies of ~34.5 THz are well outside the strong absorption range of the surrounding water molecules (the fingerprint peaks are approximately between 0 and 30 THz) [24]. The THz photons (close to 34.5 THz) are mainly selectively absorbed by the binding pocket of NR2B due to the limited absorption by water molecules, while glutamate molecules are largely unabsorbed.

Patients with PTSD often exhibit abnormal learning and memory abilities [25]. The imaging findings revealed a noteworthy decrease in hippocampal volume among individuals diagnosed with PTSD, resulting in an impairment of its cognitive functioning [26–28]. Here, we proved a new method to improve learning and memory abilities in PTSD rats. We used THz photons to irradiate the CA3 subregion of the hippocampus in animal brains by delivering THz photons through optical fibers and cannulas into the target brain area (Fig. 1). We determined the coordinates of this area from the brain atlas as (1.5, −2.16, −3.5) and fixed the optical fiber on the brain stereotaxic device to facilitate the control of the optical fiber to accurately reach the target position. We set 3 power densities at low, medium, and high levels (22, 44, and 88 W/cm^2^).

The single prolonged stress (SPS) method is a commonly used animal model of PTSD that can not only accurately simulate the clinical symptoms of PTSD patients but also show abnormalities in the hypothalamic–pituitary–adrenal (HPA) axis. It is postulated that SPS is an appropriate animal model of PTSD [29,30]. We developed an animal model of PTSD using SPS method and conducted experiments following the procedure shown in Fig. 2B. To investigate the effects of THz photon irradiation on cognitive impairment induced by SPS, we performed a novel object recognition test, a shuttler box test, a Y-maze test, and a Barnes maze test reflecting hippocampus-dependent learning and memory abilities. The results showed that the proportion of time animals in the model group spent exploring new objects was significantly reduced, while THz photon (34.5 THz) irradiation increased the proportion of time spent exploring new objects in PTSD rats (Fig. 2C, increased by about 10.0%, *P* < 0.001). However, not all irradiated rats performed this way. This effect could only be achieved under certain conditions (44 W/cm^2^). For the shuttler box test, after training, we recorded initiative and passive avoidance times and calculated the accuracy as a reflection of learning and memory function. The results showed that the accuracy of PTSD rats was significantly lower than that of healthy animals, and THz photon irradiation improved the accuracy by ~100% (Fig. 2D, *P* < 0.001). This means that THz photon (34.5 THz) irradiation improved working memory in PTSD rats. In these 2 tests, all rats in the sham group showed similar behaviors to the rats in the model group (*P* = 0.13.5; *P* = 0.2503), indicating that the method of embedding cannulas to establish THz photon radiation did not affect the learning and memory ability of rats. Subsequently, we used the Barnes maze test and Y-maze test to further explore the effect of THz photon irradiation on the learning and memory ability of animals. The results showed that in the Barnes maze, the numbers of field entries in the model group were significantly higher than those in the healthy-control group and lower than those in the medium-power-density group (Fig. 2E; was about 5 times lower, *P* = 0.001; *P* = 0.0005). Similar results were observed in the Y-maze test, where the proportion of time for PTSD animals to explore new arms was significantly reduced, while the proportion of time for animals to explore new arms significantly recovered after medium-power-density THz irradiation (Fig. 2F; increased by about 40%, *P* = 0.0329; *P* = 0.0357). These 4 behavioral experiments all demonstrated that 34.5-THz photon irradiation of the hippocampal CA3 subregion could effectively recover the learning and memory abilities of PTSD animals.

### Decreased glutamate content and increased phosphorylated NR2B in the CA3 subregion

**Fig. 3.**
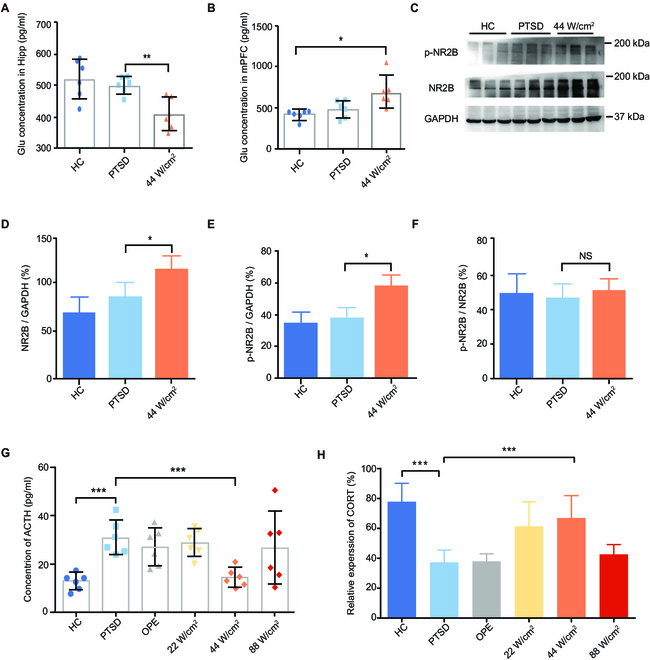
In vivo effect of THz photon irradiation on the expression levels of NR2B, p-NR2B, and HPA axis hormones. (A and B) Effect of THz photons (34.5 THz) irradiation on the concentration of glutamate in CA3 subregion and mPFC, detected by ELISA, Glu, glutamate. (C to F) Effect of THz photon irradiation on the repression of NR2B and p-NR2B. THz photons could effectively increase the expression levels of NR2B and p-NR2B. (G and H) Changes of ACTH and CORT concentration in peripheral blood. (All data shown as means ± SD, *P < 0.05; **P < 0.01; ***P < 0.001.)

Based on the results of behavioral, we further investigated the brain regions after THz photon irradiation, including the detection of glutamate concentration in the hippocampal CA3 subregion tissue and the assessment of NR2B phosphorylation levels. First, we detected the concentration of glutamate (glu) in the hippocampal CA3 subregion tissue and medial prefrontal cortex (mPFC) tissue. The results showed that there was a significant decrease in glu concentration in the hippocampal CA3 subregion tissue but an increase in glu concentration in the mPFC tissue (Fig. 3A and B; *P* = 0.004; *P* = 0.0385). Glutamate bound to NR2B promoted the phosphorylation level of NR2B. Therefore, detecting the phosphorylation level of NR2B could serve as an indicator of the binding ability between glutamate and NR2B. The western blotting results showed that there was increased phosphorylated NR2B (p-NR2B, increased by about 50%) and total NR2B (increased by about 40%) (Fig. 3, C to F; *P* = 0.0439; *P* = 0.0115) in the hippocampal CA3 subregion. This result suggested that the decreased glutamate concentration in the hippocampal CA3 subregion was caused by the enhanced binding of glutamate to NR2B by THz photon irradiation. Moreover, glutamate binding to NR2B promoted the phosphorylation of NR2B.

Furthermore, patients with PTSD often exhibit alterations in HPA axis activity, and changes in cortisol are associated with symptoms of PTSD [31]. We detected changes in hormone levels associated with the HPA axis in the peripheral blood. The results showed that the adrenocorticotropic hormone (ACTH) levels of PTSD rats were significantly increased by approximately 200% and were restored to normal levels after THz irradiation (Fig. 3G; *P* = 0.0002; *P* = 0.0007). The results showed a significant decrease in corticosterone (CORT) levels in peripheral blood in the model group, and THz irradiation also restored CORT levels (Fig. 3H; *P* < 0.0001; *P* = 0.0016).

### THz photon irradiation could reduce the binding affinity of glutamate to NR2B

**Fig. 4.**
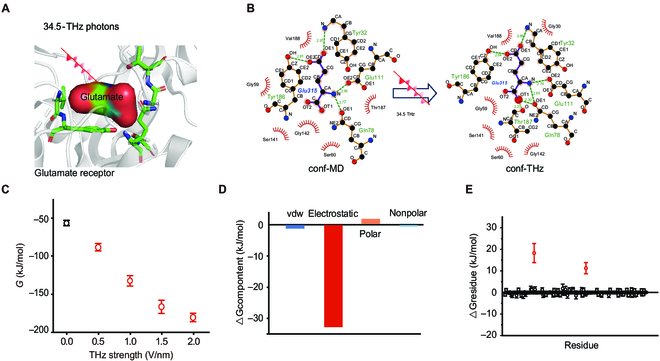
The effects of 34.5-THz photons on the activity of glutamate on the NR2B were investigated by using molecular dynamics stimulation. (A) Schematic representation of the effect of THz photons on glutamate with NR2B receptors. The central sphere represents the glutamate molecule (shown as a surface), the green amino acid represents the receptor binding pocket with glutamate, and the white background represents the glutamate receptor. (B) The mode of binding between glutamate and the receptor (H-bond network and hydrophobic interaction) before (left section) and after THz photon exposure (right section). (C) The binding energy (*G*) of glutamate to the NR2B receptor with intensities of 0 to 2.0 V/nm and a frequency of 34.5 THz. (D) Variations in binding energy due to contributions from hydrogen bonding and hydrophobic interactions. (E) Difference in the change in the binding energy and the distribution of the amino acids in the NR2B receptor. THz photons were added to the model, and the frequencies were set to 34.5 THz with an intensity of 0.5 V/nm.

To investigate the efficiency of THz photons (34.5 THz) on the utilization of glutamate molecules by the NR2B receptor, we compared the effect of THz photons stimulation on the binding free energy (*G*) and the binding interaction modes between NR2B and glutamate using molecular dynamics methods. The glutamate receptor (binding core region) forms nonbinding interactions with glutamate (surface shape) mainly through several sets of key amino acids and has a typical pocket structure, as shown in Fig. 4A. At room temperature (300 K), after kinetic simulations for ~100 ns to reach thermodynamic equilibrium, 4 stable hydrogen bonds form between amino acids Tyr186, Gln78, Glu111, and Tyr32 within the binding pocket, and 6 sets of amino acids form hydrophobic interactions. Interestingly, as shown in Fig. 4B, we found that 2 new hydrogen-bonding interactions between amino acids Gln78 and Thr187 were added after applying THz photons (frequency *ν* = 34.5 THz, intensity *A* = 0.5 V/nm), which resulted in a decrease in the free energy of glutamate receptor-glutamate binding by ~31.98 kJ/mol, indicating that the THz photons enhanced the binding capacity between NR2B and glutamate (Fig. 4C). In addition, we found that the enhancement of the binding capacity by THz photons increases with increasing intensity A from 0 to 2.0 V/nm. Furthermore, we calculated the energy contribution to the difference in binding free energy (Δ*G*) before and after THz photon exposure and found that it was mainly the electrostatic component that produced a change of ~32.4 kJ/mol, which was related to that of the hydrogen-bonding effect in Fig. 4D. We finally also assigned contributions to the binding free energy differences (Δ*G*) of the constituent amino acids of the NR2B receptor and found that it was mainly the amino acids Gln78 and Thr187 that produced relatively large changes in Δ*G* (Gln78) = 18.27 and Δ*G* (Thr187) = 11.27 kJ/mol, respectively (Fig. 4E). Therefore, our simulation suggests that THz photons (34.5 THz) have resonance absorption effects on the binding pocket of the NR2B receptor and then enhance the utilization of glutamate.

### No thermal effect was generated during THz photon irradiation

**Fig. 5.**
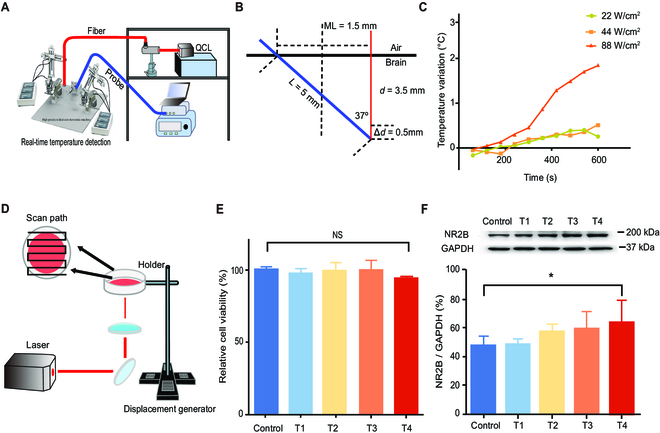
Temperature change after THz photon irradiation and no neuro injury in vitro. (A) Schematic diagram of the temperature test. (B) Schematic diagram of the temperature-sensitive probe and fiber insertion mode. Red line means the fiber, blue line means the lower edge of the temperature-sensitive probe. *d* = 0.5 mm means the depth of the fiber inserted, Δ*d* = 0.5 mm means the diameter of the temperature-sensitive probe. (C) Temperature change after THz photon irradiation. (D) Schematic diagram of the cell irradiation. (E) Relative cell activity after 24 h of THz photon irradiation, detected by MTS kit. (F) In vitro effect of THz photon irradiation on the expression of NR2B. T1: 53.6 THz-3.6 W/cm^2^; T2: 53.6 THz-6.6 W/cm^2^; T3: 34.5 THz-3.6 W/cm^2^; T4: 34.5 THz-6.6 W/cm^2^. (All data shown as means ± SD, *P < 0.05.) NS, not significant.

Under different power densities, heat may be generated by irradiation. We used a temperature-sensitive probe to detect the temperature in the irradiated region. We inserted the optical fiber into the brain at a depth of 3.5 mm and inserted the temperature-sensitive probe into the brain at a 37° angle with the optical fiber at a depth of 5 mm. The end of the probe was located 0.5 mm from the end of the optical fiber (Fig. 5A and B). The measurement results showed that when the laser started to irradiate, the tissue temperature began to rise, and within 10 min, the temperature rise in the low and medium-power-density groups was not significant (Δ*T*1 = 0.7 °C, Δ*T*2 = 0.6 °C), while the temperature increase in the high-power group was more significant, reaching 2 °C. Referring to the research results of predecessors, under irradiation with visible light or mid-infrared light, the temperature of the brain will increase to a certain extent (~2 °C), which is similar to our results (Fig. 5C), and the increase in this degree will not have an effect, inhibitory or otherwise, on the brain [16,32–34]. From this, we obtained a method that can perform THz photon irradiation on specific brain regions of animals, and this method has no significant thermal effect.

### In vitro experiment showed that THz photon irradiation improved the relative expression of NR2B without nerve injury

We also investigated whether THz photon irradiation causes damage to neurons. We designed a light path for neuronal irradiation (Fig. 5D). The beam emitted by the laser was reflected by a total mirror, changing its direction vertically upwards. Then, a lens was used for focusing, and a horizontal scanning displacement platform was placed at the focal point. The IBIDI dish with cultured neurons was placed here for scanning and irradiation. Four sets of irradiation parameters were set: 53.6 THz-3.6 W/cm^2^, 53.6 THz-6.6 W/cm^2^, 44.5 THz-3.6 W/cm^2^, and 44.5 THz-6.6 W/cm^2^. After 24 h of irradiation, an MTS (3-(4,5-dimethylthiazol-2-yl)-5-(3-carboxymethoxyphenyl)-2-(4-sulfophenyl)-2H-tetrazolium, inner salt) reagent kit was used to detect cell activity. The results showed that there was no change in cell viability in any of the 4 groups (Fig. 5E). We then demonstrated the variation in NR2B expression after irradiation by THz photons. The results showed that 34.5 THz-6.6 W/cm^2^ THz photon irradiation increased the expression of NR2B; however, changing the photons length of power did not achieve this effect (Fig. 5F; *P* = 0.0447). The results indicated that THz photon (34.5 THz) irradiation can increase the expression of NR2B in neurons cultured in vitro. This result indicated that THz photon irradiation did not cause damage to neurons. Thus, these conditions of THz photon irradiation were neuro-safe.

## Discussion

The hippocampus is a crucial structure in the brain and is mainly composed of the dentate gyrus and cornu ammonis (CA3, CA2, and CA1) subregions [35], and memories initially formed in the hippocampus gradually stabilize to the cortex over weeks to months for long-term storage [36]. The hippocampal CA3 subregion plays a crucial role in the storage, retrieval, and processing of both spatial and nonspatial information [37]. Furthermore, NMDARs play a crucial role in the CA3 subregion. Activating NMDARs not only enhances the interaction between the CA3 region and other subregion, such as the mossy fibers, as well as synaptic plasticity in the CA3 subregion's circuitry [38,39], but also contributes to the learning and memory abilities of the hippocampus within the CA3 subregion [37,40].

In this research, we presented an innovative methodology called DBTS with the advantages of characteristic THz interaction with biomolecules compared to the optogenetics technique requiring exogenous genes, and more precise stimulation compared with the dispersion of electric and magnetic technique. By precisely coupling the THz photons emitted from QCL into a low-loss THz fiber, a stereotaxic apparatus was employed to insert the fiber through an implanted metal tube, reaching the designated deep brain region with utmost precision. It is discovered that 34.5-THz photon irradiation of the CA3 subregion of the hippocampus could improve cognitive function in PTSD rats in vivo. Further investigations revealed that this improvement in cognitive function was due to irradiation promoting the binding of glutamate to NR2B, thereby increasing the phosphorylation level of NR2B. NMDARs in the CA3 subregion are directly related to associative memory [41], and increasing the activity of NR2B could help enhance the function of the CA3 region.

Previous studies have found that 34.88 THz, which is very close to the frequency we used (34.5 THz), plays an important role in neuromodulation and can modulate the activity of potassium ion channels in the pretectal nucleus lentiformis mesencephali, guiding behavior [42]. In our research, we focused more attention on NMDARs, which are ion channels permeable to Na^+^, K^+^, and Ca^2+^ [11]. Different subtypes of NMDARs have similar permeability to Na^+^ and K^+^, while the NR2 subtype has a stronger permeability to Ca^2+^ [43]. The binding of glutamate with NR2B plays an important role in regulating ion channel opening. Therefore, our study focused more on investigating the effects of THz photons exposure on other sites of ion channel proteins, specifically the binding ability of glutamate with NR2B. In our study, we found that THz photon irradiation does enhance the binding ability of glutamate with NR2B.

Compared to other diseases that can cause cognitive impairment, such as Alzheimer's disease and Parkinson's disease, the causes of cognitive dysfunction in these diseases are more complex [44–46]. Although PTSD is an irritability disorder and has many clinical symptoms, some theories propose that these clinical symptoms are mainly due to abnormal memory and inability to concentrate. According to the imaging research results, although there is dysfunction in multiple brain regions of PTSD patients, the main reason for cognitive dysfunction is the reduction in hippocampal volume. Therefore, stimulating the hippocampus of PTSD animals to restore or enhance the function of the hippocampus is expected to improve the cognitive function of these animals. In vivo experiments result in this study indicated that different power densities of THz photon irradiation were administered to assess the impact on situational memory, working memory, and spatial memory in animals. It was observed that only at the power density of 34.5 THz-44 W/cm^2^ exerted a significant effect. The results showed that the effect of THz photon irradiation was dose-dependent. Under low-power-density conditions (22 W/cm^2^), the irradiation has no effect on the behavior of TPSD animals. However, with increased power density (44 W/cm^2^), the cognitive function of animals with PTSD was significantly enhanced. It is important to note that excessive increase in power density (88 W/cm^2^) does not improve cognitive function. This is possibly attributed to the thermal effect due to high energy density, which related to accompanying damage. This side effect is challenging for most researchers to address [14]. Therefore, it is crucial to control the power density during THz photon irradiation, as higher power does not necessarily yield better results and may instead cause damage.

Interestingly, after THz irradiation, the hormonal imbalance of the HPA axis in PTSD animals also improved. We speculate that there are 2 reasons for the occurrence of this phenomenon. One of them involves the treatment leading to decreased irritability via enhancing cognitive function and emotion in the animals, thus restoring hormone levels. Another explanation involves another function of the hippocampus, endocrine function. However, whether THz photon irradiation can influence endocrine function is not involved in this study.

In summary, we provided a novel approach that can improve cognitive function in PTSD animals, which was distinguished from previous pharmacological treatments or cognitive restorative treatments [44,45]. Direct irradiation of the CA3 subregion with THz photons enhanced glutamate binding to NR2B in this subregion, enhanced the phosphorylation level of NR2B, and thus enhanced the role of glutamate transmitters and NR2B receptors in learning and memory function. This method of radiation is a nonthermal method. Together, our results illustrate that THz photon irradiation of the CA3 subregion of the hippocampus can restore cognitive function in PTSD animals, providing a potential therapeutic strategy for the treatment of this disease.

## Materials and Methods

### Light source and in vivo THz photon irradiation

In the in vivo experiments, the laser used was a 34.5-THz frequency QCL (frequency 34.5 THz, pulse duration 2 μs, repetition rate 200 kHz, duty cycle 40%). Once the THz photons emitted by the QCL passed through the THz lens and was focused, it was then coupled into the fiber by using a 3-dimensional mobile platform. The optical fiber was standard polycrystalline infrared AgCl:AgBr polycrystalline fiber (PIR240/300, art photonics) with a core diameter of 240 ± 15 μm, a clad diameter of 300 + 0/−15 μm, and a numerical aperture of 0.3 ± 0.03. To precisely target a specific brain area, a stereotaxic apparatus was utilized to implant a metal tube at a predetermined position (±1.5 mm, −2.16 mm, −3.5 mm, determined according to the rat brain atlas) in the animal's head. The metal cannula was purchased from RWD Life Science Co., Ltd (RWD, 62049), and the inner and outer diameters is 0.45 and 0.64 mm, respectively. After the cannula was fixed, the optical fiber was then inserted into the metal tube, guided by the stereotaxic apparatus, to reach the designated brain area (3.5 mm deep into the brain). This setup enabled precise irradiation to be performed on the specific brain region with accuracy.

### Western blotting and cell activity detection

Tissue from the CA3 subregion was isolated for assaying. Tissues and cells were lysed by radioimmunoprecipitation assay lysis buffer, and total protein was extracted for western blotting experiments. The primary antibodies were NR2B (Cell Signaling Technology, 14544s) and glyceraldehyde phosphate dehydrogenase (Cell Signaling Technology, 5174s), and the secondary antibody was horseradish peroxidase-linked anti-rabbit immunoglobulin G (Cell Signaling Technology, 7074s). After 24 h of irradiation, the cell activity was detected using the MTS reagent kit (Promega, G3581).

### Animals and model

Sprague-Dawley rats were purchased from Sibeifu (Beijing) Biotechnology Co., Ltd. All rats were randomly divided into cages and fed freely every day, with 12 h of light exposure per day. After 1 week of adaptive feeding, the experiment was conducted. In the experiment, animals were divided into a healthy-control group, a model group, a sham surgery group, a low-power-density group (22 W/cm^2^), a medium-power-density group (44 W/cm^2^), and a high-power-density group (88 W/cm^2^). Except for the healthy-control group, all other groups underwent modeling operations. Modeling was performed by adopting the SPS method. The animal was first restrained for 2 h, followed by forced swimming for 10 min. After being removed and wiped dry, each animal was anesthetized with isoflurane. After the animal completely lost consciousness, it continued to be anesthetized for 15 min. Then, the animal was placed back in the breeding cage and allowed to wake up. After the modeling was completed, the animals were kept undisturbed for 6 days. After the establishment of the animal model, the animals were subjected to tube embedding surgery, and the coordinates of the hippocampal CA3 area (1.5, −2.16, −3.5) were determined based on the brain atlas. Double cannulas were customized according to these coordinates, and the cannula was inserted at the predetermined location using a brain stereotaxic locator. The cannula was fixed with dental cement. Three days after surgery, THz radiation treatment was started, and the animals were anesthetized for radiation. Each side of the hippocampus was irradiated for 10 min once a day for 3 days. Behavioral experiments were conducted on animals after irradiation. All animal experiments had undergone animal ethics review and approved by Institutional Animal Care and Use Committee (review number: IACUC-DWZX-2021-737).

### Behavior test

The learning and memory abilities of the animals were tested by behavioral experiments, including novel object detection, shuttle box experiments, Barnes maze, and Y-maze experiments. The novel object detection experiment tests the situational memory ability of animals. In the novel object detection experiment, first, the animals were placed into the experimental box to adapt for 5 min. Then, 2 objects were placed into the experimental box, and the animals were familiarized with them for 5 min. After 2 h, one of the objects was replaced with a new object, and the animal was placed into the box for testing. A camera was used to record the animal’s behavior, and RWD analysis software was used for analysis.

The shuttle box experiment tests the working memory ability of animals. In this experiment, the animals were first given acousto-optic combined stimulation and then given acousto-optic stimulation for 10 s, followed by electric stimulation for 10 s. The 2 stimuli were combined for training. The behavior of animals escaping to the safety area after receiving acousto-optic stimulation was recorded as active escape, and the behavior of animals escaping to the safety area after receiving electric stimulation was recorded as passive escape. The animals were trained 20 times a day, with a 30-s interval between each training, and the number of active evasions was observed. When the number of active evasions of healthy animals reached at least 18, testing of the animals began. Finally, the active avoidance rate of animals was recorded as the evaluation standard of the working memory ability of the animals.

The Barnes maze is an improved version of the water maze experiment, which tests the spatial memory ability of animals. The experimental maze consisted of a circular platform with a circle of holes with a diameter of 10 cm around the platform. One of the holes is fixed as the target hole. The animals were first placed in the maze to adapt for 5 min, and after an interval of 2 h, the animals were placed in the maze to train them to find the target hole; the time to find the target hole and the number of times exploring nontarget holes were recorded. The animals were trained twice a day, and when the number of times the healthy-group animals explored nontarget holes was less than 2, training was stopped, and testing was started.

### Neuron culture and irradiation

Eighteen-day-old pregnant mice were purchased from Sibeifu Biological Co., Ltd., and their fetuses were used for primary neuronal culture. IBIDI Petri dishes were used to culture the isolated neurons. First, Dulbecco's Modified Eagle Medium/Nutrient Mixture F-12 medium (Gibco, 11330057) containing 10% fetal bovine serum (Gibco, 10099141C) was cultured in a CO_2_ incubator for 4 h and then changed to neurobasal medium containing B27 (Gibco, 17504001), glutamine (Gibco, 25030081), and penicillin-streptomycin solution (Gibco, 15140122). The medium was changed every 2 days. After 7 d of cell culture, irradiation began. The lighting conditions were 53.6 THz-3.6 W/cm^2^, 53.6 THz-6.6 W/cm^2^, 34.5 THz-3.6 W/cm^2^, and 34.5 THz-6.6 W/cm^2^. The movement speed of the displacement table was 0.2 mm/s, and the scanning area was a square with a side length of 4.4 cm. It was determined that all areas with cells were irradiated. After irradiation, the cells were placed in an incubator and cultured for 24 h.

### Detection of HPA axis hormone and glutamate levels

In PTSD animals with HPA axis disorder, ACTH and CORT were selected for detection (Cloud-clone, CEA836Ra; CEA540Ge). Under anesthesia, peripheral blood was extracted from the animal using the abdominal aorta blood collection method, and plasma was separated for use in the experiment. An enzyme-linked immunosorbent assay (ELISA) detection kit was used to detect ACTH and CORT levels. In addition, ELISA kits were also used to detect Glu levels in hippocampal CA3 tissue (Cloud-clone, CES122Ge). The animal’s hippocampal CA3 tissue was isolated for homogenization, and ELISA experiments were performed on the tissue homogenates.

### Modeling and parameterization

Our simulations were designed to investigate the molecular dynamics process of glutamate utilization by the NR2B receptor and to compare the binding efficiency supplied by THz photons. Our model is based on the NR2B receptor core model [24] (Protein Data Bank ID: 1us4) and is initially placed in the center of a cubic simulation box with a side length of 8.0 nm. In addition, the simulation box was filled with 14,515 water molecules (SPCE model), 41 potassium ions, and 45 chloride ions (i.e., salt concentration: 0.15 M) to maintain electrical neutrality of the whole system. We used the Charmm-36 force field and periodic boundary conditions [47], the Ewald connected element algorithm for electrostatic treatment [48], and the Velocity-Verlet algorithm [49] for solving the equations of motion. The truncation of the Lennard–Jones interaction and the real space part of the Ewald sum were 1.0 nm, and the convergence factor of the Ewald sum was 1.20 nm. The simulated system undergoes temperature and pressure equilibration at room temperature (300 K), resulting in the complete dissolution of water and ions around the protein to achieve a dynamic steady state. To include the effect of THz photons on the NR2B receptor, THz photons (close to 34.5 THz) were added to the whole simulated system. Since the intensity ratio of the electromagnetic component of the photons is equal to the speed of light, THz photons can be described by the equation **E**(t) = A*u*cos(*ωt*+*φ*) [50], where *A* denotes the maximum amplitude of the electric field, which determines the intensity of the electric component of photons, *ω* is the angular frequency, and **u** and *φ* denote the polarization direction and its phase, which are set to (0,0,1) and 0, respectively. The frequency of photons, *ν*, is related to angular frequency *ω* by the formula *v* = *ω*/2π. In this way, THz photons are involved in molecular dynamics simulations through electric field forces associated with **E** and the charges on all the atoms.

### Calculation of the absorption spectrum

We simulated the binding pocket of the NR2B receptor and the absorption spectrum of glutamate on the basis of the molecular dynamics approach of the classical GROMACS code [51]. The absorption spectra (Fig. 2A) were calculated from the Fourier transform of the total charge current velocity autocorrelation function of the simulated system [52]. We set the time interval for the sampling of the spectra to ~1 fs and the total sampling time to 50 ps. The absorption spectra were calculated on the basis of the Fourier transform of the autocorrelation function of the total charge current: *J*(*t*) = Σ_i_q_i_v_i_(*t*) [52]^,^ where *q_i_* is the charge of the *i*-th atom and *v*_i_(*t*) is the velocity of the *i*-th atom at time *t*. Note that the 4 residue sets that were uniquely identified as key amino acid residues of the protein pocket, Tyr32, Gln78, Glu111, and Tyr186, were used in the calculation of the binding pocket absorption spectra.

### Calculation of the binding free energy

In addition, the following 4 terms can be used to calculate the free energy of binding (𝐺_binding_): between glutamate and the NR2B receptor in solution: 𝐺_binding_ = 𝐸_vdw_ + 𝐸_elec_ + 𝐺_polar_ + 𝐺_nonpolar_, i.e., van der Waals free energy (𝐸_vdw_), electrostatic free energy (𝐸_elec_), polar or electrostatic solvation free energy (𝐺_polar_), and nonelectrostatic solvation free energy (𝐺_nonpolar_) [53–56]. The g_mmpbsa tool [55] was used here to assess 𝐺_binding_ based on simulated trajectories. Using a sampling interval of 0.5 ns, after simulating the system time for 50 ns to reach equilibrium, we continued the run for 10 ns to calculate the binding free energy, and the average binding free energy over time is calculated based on the bootstrap method. g_mmpbsa also calculates the contribution of residues to the total binding energy and identifies ligand–receptor binding at key residues in ligand–receptor binding.

### Statistical method

The experimental data in this article are all represented as the mean ± SD, and *t* test or one-way analysis of variance (clearly provided in the figures) using GraphPad Prism 6.0 software were used for difference testing.

## Data Availability

Data available on request from the authors. The data that support the findings of this study are available from the corresponding author, C.C., upon reasonable request.
